# Metagenomic identification of Acanthamoeba Rhysodes in chronic skin lesion: Case report and literature review

**DOI:** 10.1016/j.jdcr.2026.04.069

**Published:** 2026-05-11

**Authors:** Jaime David Acosta-España, Jenny Belén Altamirano-Jara, Andrés Herrera-Yela, Francisco Estrella, Santiago Palacios

**Affiliations:** a Clinical Microbiology and Infectious Diseases, Health Sciences Faculty, Research Group of Emerging and Neglected Diseases, Universidad Internacional SEK (UISEK), Quito, Pichincha, Ecuador; b Clinical Medicine, Ribeirão Preto Medical School (FMRP), University of São Paulo, Ribeirão Preto, Sao Paulo, Brazil; c School of Medicine, Pontificia Universidad Católica del Ecuador, Quito, Pichincha, Ecuador; d Centro de Investigación para la Salud en América Latina (CISeAL), Pontificia Universidad Católica del Ecuador, Quito, Pichincha, Ecuador; e Clinical Dermatology Department, Centro de la Piel (CEPI), Quito, Pichincha, Ecuador; f Dermatology Department, Veris, Quito, Pichincha, Ecuador; g Experimental and Applied Biomedicine Research Group, Health Sciences Faculty, Universidad Internacional SEK (UISEK), Quito, Pichincha, Ecuador

**Keywords:** acanthamoebiasis, case report, diagnostic errors, Ecuador, immunocompetent host, metagenomics, skin ulcer

## Introduction

Chronic ulcerative skin lesions present a significant diagnostic challenge, particularly when conventional first-line treatments fail. The differential diagnosis is broad, including infectious, neoplastic, and inflammatory conditions. Cutaneous acanthamoebiasis, an infection caused by free-living *Acanthamoeba species*, is an extremely rare cause of such lesions, especially in immunocompetent individuals.^1^ *Acanthamoeba* is more commonly associated with keratitis or granulomatous amebic encephalitis, the latter of which can be heralded by a skin lesion.^2^

Traditional diagnostics, including histopathology and culture, can be non-specific or misleading. Metagenomic next-generation sequencing (mNGS) offers an unbiased, hypothesis-free approach that enables comprehensive detection of microbial deoxyribonucleic acid (DNA) from a single sample.^3^ We present a case of a refractory, chronic cutaneous ulcer in an immunocompetent male, whose lesion was a potent clinical and histological mimic of tuberculosis. This led to a 3-year diagnostic odyssey, which was resolved after mNGS identified *Acanthamoeba rhysodes* where conventional methods had failed.

## Case report

### Patient information

**Fig 1:**
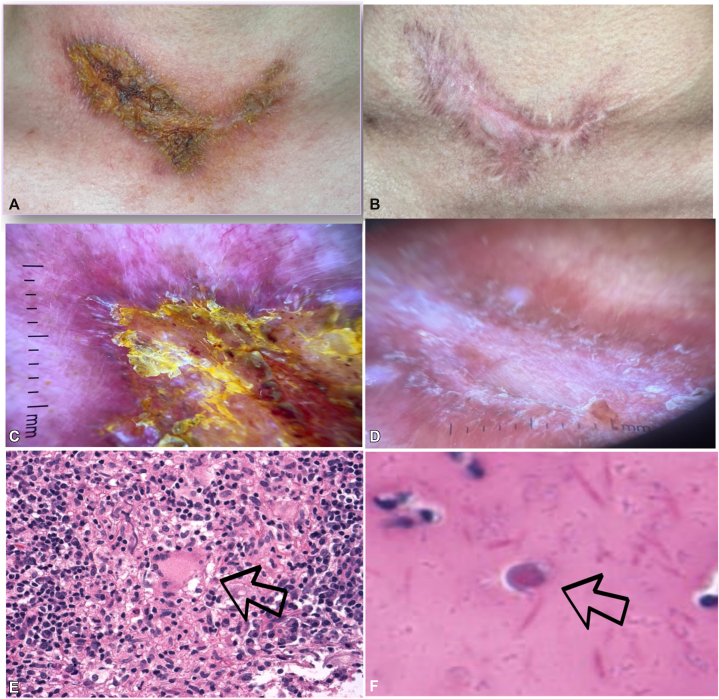
Clinical, dermoscopic, and histopathologic features of cutaneous *Acanthamoeba*. **A,** Clinical presentation at the anterior cervical base (suprasternal notch): an irregular, indurated erythematous plaque (∼10 × 5 cm) featuring a macroscopic central ulceration covered by a thick, adherent meliceric-like serosanguinous crust prior to treatment. **B,** Clinical resolution at the same site after completion of targeted therapy, demonstrating complete re-epithelialization with residual fibrotic scarring. **C,** Dermoscopic view (April 29, 2025): Nonmelanocytic lesion exhibiting a peripheral erythematous base, linear white streaks at the outer border, a central yellow-golden crust, and scattered pinpoint microhemorrhages lacking a specific vascular pattern. **D,** Dermoscopic view (September 20, 2025): healed lesion characterized by central structureless white areas (fibrosis) and absent ulceration. **E,** Histopathology (Hematoxylin–Eosin stain, 40× magnification): Incisional biopsy encompassing the papillary and mid-reticular dermis. The tissue exhibits a suppurative granulomatous dermatitis with dense nodular infiltrates of histiocytes and multinucleated giant cells (*arrow*) surrounding central foci of liquefactive necrosis with neutrophilic debris. **F,** Histopathology (Ziehl–Neelsen stain, 100× oil immersion): Targeted re-examination revealing a specific *Acanthamoeba trophozoite* (*arrow*) characterized by a prominent central karyosome and surrounding clear halo, highlighted within the dermal granulomatous infiltrate.

A 31-year-old immunocompetent man from Ecuador presented with a chronic, irregular ulcerative plaque (∼10 cm) located at the anterior cervical base, progressing over 3 years (Fig 1, *A*). His occupation in the petroleum industry involved frequent travel to jungle areas (Shushufindi and Coca). His epidemiologic history was notable for past frequent swimming in a river in Shushufindi, which was a risk factor when he lived in the Amazon region. He had since moved to Quito for treatment and avoided such environmental water exposure. He had no known comorbidities or history of immunosuppression.

### Clinical findings and diagnostic assessment

Over the 3-year course, the patient received multiple empirical treatments without clinical improvement, including systemic antimonials (13 doses) for suspected leishmaniasis, and a 3-month intensive phase regimen (rifampin, isoniazid, pyrazinamide, and ethambutol) for presumed cutaneous tuberculosis.

Physical examination revealed a V-shaped (∼10 × 5 cm), indurated erythematous plaque. The ulcerated center was covered by a thick, adherent yellow-golden (meliceric crust) crust (Fig 1, *A*). Dermatoscopy revealed a non-melanotic lesion with peripheral erythema, white linear areas, and scattered punctiform microhemorrhages (Fig 1, *C*). No lymphadenomegalia was present. A chest X-ray was unremarkable.

A 5-mm incisional fusiform biopsy was performed, encompassing the epidermis down to the deep reticular dermis. Initial tissue cultures were positive only for *Candida parapsilosis*, considered a possible contaminant. Standard histopathology (hematoxylin–eosin stain) revealed a suppurative granulomatous dermatitis characterized by dense nodular infiltrates of histiocytes and multinucleated giant cells, with central foci of liquefactive necrosis containing neutrophilic debris (Fig 1, *E*).

No microorganisms were visualized during the initial review. Crucially, the external pathology report mischaracterized the neutrophilic necrotic foci as “caseous,” resulting in an erroneous diagnosis of cutaneous tuberculosis and triggering the ineffective anti-tubercular therapy. Importantly, special stains, including Grocott-Gomori and Ziehl-Neelsen, were negative for fungal or mycobacterial elements.

Given the nonconclusive conventional tests and failure of empirical therapies, a repeat incisional biopsy was clinically indicated for histologic reevaluation. Concurrently, a portion of this new specimen was secured as fresh, sterile, unfixed tissue for metagenomic amplicon sequencing (Illumina MiSeq) targeting bacterial 16S ribosomal RNA (rRNA), fungal internal transcribed spacer, and parasitic 18S rRNA genes. Utilizing fresh tissue for this molecular analysis ensured optimal nucleic acid integrity, avoiding the DNA cross-linking and fragmentation artifacts typically associated with formalin fixation.

The bioinformatic analysis was performed on the Galaxy (AU Server) platform, with sequences classified against the SILVA v138 (16S rRNA), UNITE 8.3 (internal transcribed spacer rRNA), and nucleotide database (18S rRNA) databases, last updated Q2/2025. To ensure accuracy, DNA-free water and a sterile swab were processed simultaneously as negative controls, which yielded no significant findings. A ZymoBIOMICS Microbial Community DNA Standard was processed as a positive control, performing to specification.^4^ The analysis identified 3 key organisms:

1. Parasite (18S): *Acanthamoeba rhysodes* (67% relative abundance)
2. Bacteria (16S): *Staphylococcus aureus* (60% relative abundance)
3. Fungus (internal transcribed spacer): *Candida orthopsilosis* (9% relative abundance)

The *Candida* finding was consistent with the initial culture; however, its low relative abundance (9%) supported its role as a colonizer. In contrast, *Acanthamoeba rhysodes* was the dominant parasitic organism. Prompted by this high-abundance mNGS identification, a focused re-examination of the hematoxylin–eosin stain slides was conducted. This targeted review successfully identified specific amoebic structures consistent with *Acanthamoeba* within the granulomatous inflammation, which had been overlooked during the initial, nondirected analysis (Fig 1, *F*).

Therapeutic intervention and follow-up based on the mNGS-guided diagnosis of a co-infection, the patient was started on a long-term course of itraconazole 200 mg VO bis in die, twice daily, which was continued for 5 months. For the *S*. *aureus* co-infection, he received trimethoprim/sulfamethoxazole 800/160 mg VO bis in die, twice daily for 15 days. The patient showed progressive healing, but at the 2-month follow-up, a bacterial flare was suspected, and a second 15-day course of trimethoprim/sulfamethoxazole was administered. The patient was followed for a total of 6 months. The resolution was progressive, with the lesion achieving complete re-epithelialization by 5 months, leaving a healed scar (Fig 1, *B* and *D*).

## Discussion

This case highlights the diagnostic challenge of cutaneous acanthamoebiasis, particularly its ability to mimic granulomatous diseases such as tuberculosis. Following a review of the literature, primary cutaneous acanthamoebiasis typically manifests as disseminated nodules or ulcers, predominantly affecting immunosuppressed individuals (e.g., Human Immunodeficiency Virus/Acquired Immunodeficiency Syndrome or solid organ transplant recipients).^1^^,^^5^

In immunocompetent hosts, the disease is exceptionally rare, and infections are overwhelmingly attributed to *A*. *castellanii* or *A*. *polyphaga*.^3^^,^^5^^,^^6^ To our knowledge, this represents the first documented case of *Acanthamoeba rhysodes* (a species traditionally associated with keratitis^5^) presenting as a solitary, chronic cervical ulcer mimicking cutaneous tuberculosis in a strictly immunocompetent patient.

The paucity of similar clinical reports highlights the critical role of mNGS in identifying atypical or emerging free-living amebic species that evade conventional culture.^3^ This was later refuted by negative Ziehl-Neelsen and Grocott stains and the lack of response to antitubercular therapy. Only after mNGS provided a definitive molecular target (*A*. *rhysodes*) was a specialized re-review of the hematoxylin–eosin stain slides able to correctly identify the specific amoebic structures.

While mNGS alone cannot definitively prove causation, the high relative abundance (67%) of *A*. *rhysodes*, combined with subsequent histopathological confirmation of specific amoebic structures and a plausible epidemiological link (past exposure to river water), provides a multimodal diagnosis. The therapeutic management of cutaneous acanthamoebiasis lacks standardized guidelines, particularly for rare isolates like *A*. *rhysodes*.

The selection of itraconazole 200 mg VO twice daily was based on its established *in vitro* antiprotozoal activity against free-living amoebas. Itraconazole disrupts amebic cell membrane integrity by inhibiting sterol biosynthesis, affecting both trophozoite and cyst stages.^7^ Given the chronic 3-year progression of the lesion and the deep mid-reticular dermal involvement confirmed on histology, a prolonged 5-month course was clinically necessary to achieve complete structural re-epithelialization and prevent disease recurrence.

The detection of *Staphylococcus aureus* is also significant. *Acanthamoeba* has been shown to act as a “Trojan horse” for bacteria, potentially enhancing pathogenicity.^8^^,^^9^ We acknowledge that, as a case report, the therapeutic outcome is confounded by the combination therapy. However, the caseating granulomatous histopathology (a highly atypical presentation for *S*. *aureus*) and the failure of previous treatments make *S*. *aureus* an unlikely sole pathogen. The clinical success provides the mNGS finding of a synergistic co-infection. The necessity of a second course of antibiotics at month 2 suggests the bacterial component was a stubborn co-pathogen, while the steady resolution over 5 months on itraconazole correlates with the successful treatment of the primary, slow-growing amoebic infection.^10^

In conclusion, clinicians should consider cutaneous acanthamoebiasis in the differential diagnosis of chronic, refractory granulomatous ulcers, even in immunocompetent patients, as it can be a potent mimic of tuberculosis. This case champions the use of mNGS as a powerful diagnostic tool to resolve complex odysseys in infectious disease.

### Declaration of generative AI and AI-assisted technologies in the writing process

Grammarly was used to improve the quality of English under the strict supervision of the authors.

## Conflicts of interest

None disclosed.

## References

[1] Paltiel M., Powell E., Lynch J., Baranowski B., Martins C. (2004). Disseminated cutaneous acanthamebiasis: a case report and review of the literature. Cutis.

[2] Celis V., Meza E.R., Sialer M. (2023). Amebiasis cutánea por ameba de vida libre, heraldo ignorado de encefalitis letal. Reporte de caso. Revista del Cuerpo Médico Hosp Nacional Almanzor Aguinaga Asenjo.

[3] Fan W., Li P., Wei Q. (2024). Metagenomic next-generation sequencing-assisted diagnosis of a rare case of primary cutaneous acanthamoebiasis in an HIV patient: a case report. Front Cell Infect Microbiol.

[4] Zymo Research ZymoBIOMICS^TM^ Microbial Community DNA Standard (200 ng). https://www.bioscience.co.uk/product∼788389.

[5] Marciano-Cabral F., Cabral G. (2003). Acanthamoeba spp. as agents of disease in humans. Clin Microbiol Rev.

[6] CDC (2025). Acanthamoeba infections. https://www.cdc.gov/acanthamoeba/about/index.html.

[7] Thomson S., Rice C.A., Zhang T., Edrada-Ebel R., Henriquez F.L., Roberts C.W. (2017). Characterisation of sterol biosynthesis and validation of 14α-demethylase as a drug target in Acanthamoeba. Sci Rep.

[8] Purssell A., Lau R., Boggild A.K. (2017). Azithromycin and doxycycline attenuation of Acanthamoeba virulence in a human corneal tissue model. J Infect Dis.

[9] Rayamajhee B., Sharma S., Willcox M. (2022). Assessment of genotypes, endosymbionts and clinical characteristics of Acanthamoeba recovered from ocular infection. BMC Infect Dis.

[10] Seas R.C., Bravo P.F. (2006). [Amebic granulomatosis encephalitis due to Balamuthia mandrillaris: fatal disease increasingly recognized in Latin America]. Rev Chilena Infectol.

